# Transition for Adolescents and Young Adults With Asthma

**DOI:** 10.3389/fped.2019.00301

**Published:** 2019-07-23

**Authors:** Adelaide LIndsay Withers, Ruth Green

**Affiliations:** ^1^Department of Respiratory Medicine, Perth Children's Hospital, Perth, WA, Australia; ^2^Glenfield Hospital, Leicester, United Kingdom

**Keywords:** asthma, transition, transition process, asthma phenotype, asthma management, adolescent, young adult

## Abstract

Asthma is a complex, heterogenous medical condition which is very common in children and adults. The transition process from pediatric to adult health care services can be a challenge for young people with chronic medical conditions. The significant changes in physical and mental health during this time, as well as the many unique developmental and psychosocial challenges that occur during adolescence can complicate and impede transition if not adequately addressed and managed. The transition period can also be a challenging time for health professionals to assess readiness for transition and manage some of the complications which are particularly common during this time, including poor adherence to therapy, smoking, drug use, and emerging mental health conditions. The natural history, presentation, symptoms, and management of asthma is often significantly different when comparing pediatric and adult practice. In addition, management in infants, toddlers, school aged children, and adolescents differs significantly, offering an additional challenge to pediatric physicians managing asthmatic children and young people. Despite these challenges, if the transition process for young people with asthma is planned and performed in a formalized manner, many of these issues can be addressed, allowing the transition to occur smoothly despite changes that may occur in medical and psychosocial domains.

## Introduction

Transition is “the purposeful, planned movement of adolescents and young adults with chronic physical and medical conditions from child-centered care to adult-oriented health care systems” (1). In 1993 the Society for Adolescent Medicine published standards of care for transition, the goals being to provide a transition process that was coordinated, uninterrupted, psychosocially sound, developmentally appropriate, and comprehensive (1). In 2011 the American Academy of Pediatrics (AAP) with endorsement of the American Academy of Family Physicians (AAFP) and American College of Physician (ACP) published a clinical report that described preparation for transition, the process of transition and tracking of transition as well as appropriate follow up post transition (2). This document was updated in 2018 (3, 4). The purpose of these documents was to highlight the importance of planning for transition and to provide a framework or model for transitioning adolescent and young adult patients from pediatric to adult care in a standardized and effective manner.

Asthma is the most common chronic medical condition in children, affecting over 7 billion children in the United States of America (5) and about 300 million people worldwide (6). Although the underlying pathology (reversible obstruction of the small airways) is similar in asthma at any age, there can be marked differences in presentation, triggers, phenotype and treatment when considering asthma in children, adolescents and adults. The changes that occur in an individual during adolescence include physical, emotional, and psycho-social. The influence these changes exert upon pathophysiology, presentation, prognosis, and treatment of asthma must be carefully considered during the transition from pediatric to adult health care. For example, poor adherence to treatment is very common during adolescence as there is increasing independence and individuation, as a consequence more frequent asthma exacerbations may occur. Therefore, transitioning patients with asthma must be a carefully planned process, with consideration of the significant changes in all domains of a young person's life and the effects these changes exert upon their asthma.

## Natural History of Asthma

Patterns of wheezing are often quite variable in childhood in contrast to adults where there tends to be more stability in type, frequency and triggers of exacerbations. Viral induced wheeze is extremely common in the first year of life due to small airway caliber and the presence of multiple episodes of viral induced wheeze is not necessarily an indicator of future asthma (6). It is known that wheezing in the first year of life, even if episodes are severe, does not always predict persistence of wheezing at 10 years of age (7). Toddlers and pre-schoolers who wheeze tend to either have episodic viral induced wheeze or multiple trigger wheeze (commonly associated with personal and/or family history of atopy) (8). Viral induced wheeze (and asthma) tend to improve with age and increase in size of airway caliber, however young children who wheeze with atopy and/or a family history of asthma are at risk of persistent wheeze later in childhood (6, 9–11). The Tucson Children's Respiratory Study demonstrated that persistent wheezing in childhood is likely to persist at least to early adulthood (12). Despite asthma in children being strongly linked to atopy, the association between atopy and asthma extends beyond childhood, as atopic individuals without wheeze and/or airway hyperresponsiveness at 12 years of age can still develop asthma as adults (13).

Adolescence is a time where asthma may change significantly. Studies that have followed adolescents in a longitudinal fashion until adulthood have focused on asthma remission, persistence, relapse and new onset adult asthma (6). Although cross sectional studies have demonstrated that significant changes occur in the asthmatic population in adolescents, individual factors that influence prognosis have not been identified (14). Longitudinal studies of adolescents and young adults have demonstrated that bronchial hyperreactivity as a child is a strong predictor of persistence of asthma in adult life (15–18), however these studies have not allowed description of an accurate asthma “phenotype” in adolescents and young adults (14). Interestingly, the higher incidence of asthma in boys compared to girls tends to reverse after adolescence with asthma becoming more common in women than men (11, 14, 19, 20). This may be related to male sex being a risk factor for late onset atopy and female sex a risk factor for later onset bronchial hyperreactivity (14). However, as Xuan et al. (13) point out, this observation could be also caused by a higher rate of onset of asthma in puberty in females or a lower remission rate of asthma in females after puberty. It may also reflect better response to treatment in childhood in males with increased likelihood of resolution (6).

Remission of asthma can occur during adolescence (14, 21–23), with some reporting that “most” children have remission in adolescence and early adulthood (6, 24, 25). To the contrary, Xuan et al. (13) demonstrated in their longitudinal cohort of young adults recruited at ages 8–10 that onset of wheeze during follow up was more common than remission (12.4 vs. 5.6%) and the prevalence of wheeze increased by 6% in a 10 year follow-up period. Importantly, even if asthma truly remits, it is not known if remission of symptoms is the same as resolution of underlying airway pathology (6). The observations of a longitudinal Dutch cohort suggest that underlying airway pathology does not resolve despite remission of symptoms, as asymptomatic patients still had abnormal lung function and/or persisting airway hyperresponsiveness (26).

Between 3–5% of people who wheeze in childhood continue to wheeze as adults (6, 24). As demonstrated by the Tucson Children's Respiratory Study, persistent wheezing in childhood is likely to persist into early adulthood, with airway hyperresponsiveness at 6 years of age predicting the presence of asthma at 22 years (11). Other groups have demonstrated that persistent asthma in adulthood was associated with lower lung function in childhood, persistence of airway hyperresponsiveness, atopy by the age of 13 and early smoking as a young adult (27–29). In severe asthmatic adults, 69% reported asthma symptoms were present before the age of 20 years (8).

Asthma which has resolved in childhood may relapse later in life, even if asymptomatic as adolescents or young adults (6, 24, 27, 30). Relapse of symptoms in adulthood was associated with smoking (particularly in those who were not atopic), asymptomatic airway hyperresponsiveness at 13 years of age and atopy (24, 27).

Knowledge regarding asthma with the onset in adolescence or young adulthood is limited (13). Although new onset bronchial hyperresponsiveness is unusual in adolescence, the appearance of asthma in previous well adolescents and young adults is described (13). Adult onset asthma generally has a poorer prognosis and poorer response to treatment when compared to childhood onset asthma. In a longitudinal cohort study of young adults with asthma, of those who wheezed at any time, 7.8% had developed airway hyperresponsiveness after the age of 8–12 years (13). Atopy at ages 8–12 and parental history of asthma were also predictors of late onset wheeze and female sex a predictor of late onset airway hyperresponsiveness (13). However, it has been postulated that recall of childhood symptoms as an adult may be poor, and that adult onset asthma may actually represent a relapse rather than true adult onset of asthma (6). This is supported by the findings of the Tucson Children's Respiratory Study, where 63% of participants reporting “new” symptoms as adults had already reported symptoms as children (11). The effect of cigarette smoking must also be taken into account for adults with the onset of asthma (6). In addition, new onset adult asthma may be a different phenotype as individuals are more likely to be female, obese, non-atopic and smokers (31–33).

## Pediatric, Adolescent, and Adult Asthma—Differences and Similarities

Marked differences exist when comparing asthma in children, adolescents and adults. Even when considering children with asthma as a group, significant heterogeneity exists when comparing toddlers, school aged children and teenagers. To further complicate matters, asthma during the adolescent and young adult period has unique features and from an epidemiological perspective, the transition period from childhood asthma to young adult asthma is poorly understood (14). For these reasons, asthma is best thought of as a heterogenous, complex syndrome rather than a single disease (6, 34).

Phenotyping is not commonly applied to pediatric asthma as it does not tend to be particularly useful, in contrast to adult asthma (8, 35). Phenotypes can be defined in various ways, such as disease severity, presence of atopy or inflammation, temporal patterns of symptoms, response to treatments or triggers (6). When phenotypes are applied to children with wheeze and/or asthma, children frequent move between groups when they are young (34), making phenotypes less useful for prognostication. This is not surprising given the variability in the natural history, persistence and remission/relapse of asthma during childhood and adolescence. In addition, phenotyping in adults often relies on biomaterials sampled from the lower airways, which is often not available in children (6). Phenotyping by atopic status is not useful in preschool children as presence of atopy is often unclear, there is poor correlation between atopy and wheeze in this age group and atopy does not predict response to inhaled steroids (8). When considering severe asthma, older children with severe asthma tend to be male, highly atopic and are usually not completely steroid responsive which is in contrast to severe adult asthmatics who tend to be females, atopy is less common and neutrophilic inflammation is common (8).

It is known that asthma in early life is a risk factor for asthma and COPD in later life (27, 36–38). Although children who wheeze in early infancy often don't go on to develop asthma in childhood, they too have been shown to be at risk of developing COPD in adult life (6).

Therefore, it is difficult to say how similar pediatric and adult asthma are overall, given the complexities of early wheeze phenotypes, but the demonstration that many children with severe asthma continue to have severe asthma as adults (8, 11, 39) suggests that *severe asthma* may be similar in these different age groups. Prevalence of severe asthma in childhood ranges widely depending on location from 2.1% (40) of children in Sweden with asthma to 5% (41) in the U.S.A, 8.8% in Spain (42) and 36% in Canada (43). Therefore, given the heterogeneity in early viral induced wheeze and preschool wheeze, the authors estimate that approximately 10% of cases with adult asthma and childhood asthma have the same condition.

## General Considerations for Adolescent and Young Adult Health Care

Some general considerations when seeing adolescent and young adult patients with medical conditions include seeing adolescents without their parents, increasing autonomy, confidentiality, consent, puberty/contraception/sexual health, poor adherence to medical treatment, mental health concerns, substance use and screening for risk taking behaviors (44). It is important to note that the following considerations not only apply in the pediatric health care system, but after transition to adult services. In addition, addressing these issues may prove very useful for adult physicians to help in building rapport, particularly during the first few appointments with a new patient who has just transitioned to adult services. Following the same principles of age and developmentally appropriate adolescent and young adult health care in adult services ensures continuity of care standards and demonstrates the commitment of the adult health professionals to providing individualized care that is tailored to age and maturity of the young adult.

As a pediatric patient enters adolescence, ideally at least some of the consultation should occur with the patient on their own (44). As adult patients will often be seen on their own, it is good to prepare young people to be able to provide relevant history and information for their appointment. This can be particularly challenging for pediatric physicians when they have known the patient since infancy, and are used to taking the majority of history from the parents (44). This concept can also prove very challenging to parents. Parents may worry their concerns won't be voiced by their child, that “secrets” may be withheld from them or that their child is unable to take responsibility for their own health care. Parents should be reassured that this is a normal, routine part of health care for all young people, and an excellent way to start preparing for future transition. Young people with cystic fibrosis have indicated that they wish to be seen at least partly on their own between the ages of 13–16 as they have private issues they wish to discuss with their doctor (45). Of concern, some young people indicated they were never offered the opportunity to be seen on their own (45). Gently introducing the idea of being seen alone for part of the consultation around the age of 12–13 is useful, with the parent present initially to provide any additional information about the medical concerns (while encouraging the adolescent to answer the questions), then some time with the adolescent alone to build rapport (44). After a rapport is built and confidentiality is discussed, screening for adolescent issues can occur at subsequent appointments. Explaining to the young person that seeing them alone is important as there may be health concerns they may not want to discuss with their parents present, and normalizing the process as part of becoming independent and assuming responsibility for their own health care (44). Discussing confidentiality is imperative so that parents and patients are aware of this concept and exceptions to confidentiality (discussed below).

Confidentiality is of the utmost importance when seeing an adolescent or young adult patient alone, and it must be clear that they understand the exceptions to confidentiality (if someone is hurting them, they are in danger of hurting themselves or hurting someone else) (44). It is also helpful to explain that sometimes health information will need to be shared with other members of the medical team, but that this would only happen with permission from the adolescent. It is particularly important to discuss confidentiality prior to undertaking an adolescent health screen, as information may be disclosed which would require a breach of confidentiality (for example, disclosure of sexual abuse or suicidal ideation). The need to disclose this information and break confidentiality can be extremely damaging to the therapeutic relationship if the young person feels they have been betrayed or punished for disclosing this information. Clear discussion of the limits of confidentiality is therefore essential in maintaining the therapeutic relationship.

Providing consent for medical procedures will vary based on local laws. Under common law in Australia and the UK, adolescents under the age of 16 can provide informed consent without parental knowledge if they are deemed by the treating doctor to have sufficient understanding of the proposed treatment, consequences of accepting or rejecting treatment and alternative options (44). The gravity of the situation must be considered; as clearly there is a significant difference between a 14-year-old providing consent for antibiotics for a skin infection vs. providing consent for a chemotherapy. If there is any doubt seeking legal advice and advice from an ethics committee is advisable, particularly when there may be child protection concerns, questions about capacity of an adolescent to provide valid consent in the context of intellectual disability, mental health concerns, suicidal ideation or disagreement between the adolescent and their parents/legal guardian. In addition, clear documentation of conversations and decisions about providing consent, the adolescent's understanding of risks and benefits of treatment etc. must be recorded in the medical notes.

Adolescent health screening is not only an important part of providing age appropriate medical care but can assist in establishing a rapport with a young person. This is particularly important when an adult physician is meeting an adolescent patient for the first time, as it helps to establish who the young person is, their interests, their family and living situation and any health risk factors that may be present. Given the very personal nature of some of the questions, it is best to start with topics such as whom they live with before moving to topics such as drug use and sexuality. This may need to occur over a number of visits, particularly when meeting a young person for the first time. This process is often straightforward for pediatric physicians as they will usually know the adolescent quite well, but paradoxically it can complicate health screening as young people may be more reluctant to disclose risk taking behaviors to a doctor they know well for fear of disappointing their doctor or fear of their parents being informed. Often honest responses are obtained by normalizing the process (“we ask everyone your age these questions”) and normalizing their situation (“lots of people your age drink alcohol, do your friends drink? What about you?”). A useful acronym to perform an adolescent health screen is HEARDDSS (home environment, education, activities they participate in, relationships with friends, family and opposite/same sex, drug use including smoking, alcohol and illicit drugs, depression, sexuality and suicide risk/risk taking behaviors) (44).

Generally by the time transition is occurring, adolescents will have completed growth and puberty but this is not always the case in chronic medical conditions. It is important to be sensitive as pubertal delay and short stature can be extremely distressing to young people, particularly if they look noticeably different and younger than their peers. Regular assessment of height and weight at each visit is essential and monitoring pubertal status with Tanner staging on a growth chart is most easily done by getting the adolescent to self-identify the picture that looks most like them. If significant pubertal delay and/or short stature are evident, assessment of bone age should be performed as well as screening for other medical causes of pubertal delay/short stature such as coeliac disease and thyroid dysfunction. Referral to an endocrinologist should be considered, particularly for those on high dose inhaled or oral steroids or complex medical conditions, as augmentation of puberty and/or Growth Hormone may be considered.

Following on from this, contraceptive needs should always be addressed, particularly when pregnancy may have an adverse effect on the underlying medical condition (for example cystic fibrosis and Type 1 Diabetes). Discussion about planning for future pregnancies, impact of pregnancy on the medical condition and potential side effects/contraindications of various methods of contraception should be discussed from early puberty. Consideration should be given to offering genetic counseling in inherited diseases when adolescents are in established puberty, and “safer sex” should always be encouraged to minimize risk of unwanted pregnancy and sexually transmitted diseases.

Adolescence is naturally a time of testing limits and boundaries, and often risk taking behavior such as experimentation with smoking and drug/alcohol use occurs (44). There is conflicting evidence regarding whether young people with chronic medical conditions are more or less likely than their peers to smoke or use drugs/alcohol (46, 47). Smoking can be particularly difficult to address, as adolescents will often want to experiment with their peers to fit in and minimize differences between themselves and their “well” peers. As adolescents often do not have the cognitive capacity to understand future consequences as a result of their current actions, explaining how smoking reduces lung function in the long term is unlikely to be successful in dissuading them not to smoke. Immediate consequences, particularly when related to appearance (such as yellow nails and teeth from smoking or missing out on a party as they require admission) may act as a deterrent to smoking (44).

Depression and anxiety can emerge or worsen in adolescence, particularly when there is a chronic medical condition. Mental health problems can have a negative effect on health outcomes, health related quality of life and adherence to treatment (44). Depression is common in adolescents, with the incidence of major depression estimated between 3 and 5% (48, 49), unsurprisingly with a higher incidence (up to three times) in adolescents with a chronic medical condition (48–51). Chronic medical conditions are also known to increase the risk of suicide attempts, particularly in females (52, 53). It is important to screen for and refer on to appropriate services if there are concerns about emerging depression, anxiety or suicidal ideation, and recognize how these conditions can affect medical outcomes and adherence to treatment.

Adherence to medical treatment can be particularly challenging in the adolescent period for a number of reasons. Mean adherence rates for long term treatments in adolescents with a chronic medical condition are reported between 33 and 94% (44). Adherence is known to decrease in adolescence in those with chronic medical conditions (54, 55). Adolescents naturally test limits and boundaries and wish to become independent. Often part of developing autonomy includes adolescents not wishing to be “told” what to do, and this includes taking medications, doing airway clearance, physiotherapy and attending appointments. Barriers to adherence include a wish to rebel, lack of time, school commitments, not wanting to seem different to peers, forgetting, disagreement with the physician and feeling that treatments don't work. Non-adherence to treatment can impede transition, and occasionally a young person will purposefully stop taking their treatment to delay transition (unfortunately this may have the opposite effect and the pediatric team may transition out of sheer frustration) (44, 56). Pediatric physicians may also wish to delay transition as they do not feel confident the young person is adherent enough to manage their medical condition in the adult system. Non-adherence is best addressed with patience and over time, exploring in a sensitive and non-judgemental manner the barriers to adherence. Again, it can be helpful to normalize that non-adherence is common, trying to solve the problem together by offering choices, acknowledging the treatment burden and minimizing unnecessary or duplications of treatments. Focusing on the “most important” treatment can be helpful, as well as setting review dates for treatment and setting realistic goals. In some situations, the only way forward is to continue to reassure the young person that you will continue to see them and stick with them throughout the process regardless of whether they are adherent or not. Outlining issues with adherence with the adult team is crucial so that continuity of care after transition can be assured and that patients do not “fall through the gaps” if they fail to attend future appointments with adult services. This can become a particular problem as the pediatric health care system is often more accommodating when it comes to poor attendance, whereas adult services simply may not have the capacity to repeatedly book non-attenders.

## Models of Transition

Although many different models for transition have been proposed, the Society for Adolescent Medicine position statement highlights the lack of published research comparing the different models of transition, in particular whether certain models performed better for specific medical conditions/illness severity or even if a formalized transition program actually improved health care outcomes (1). A review by Wright et al. (57) as well as the AAP report also highlighted the paucity of randomized control trials comparing different models of transition, as the majority of transition related research comprises interventional studies. Despite the existence of numerous models for transition, many share the core components of being well-prepared, starting transition early, familiarization with the adult health care system prior to transition and use of joint pediatric/adult clinics. Models draw on frameworks, concepts, and core components such as the AAP transition theory framework core principles of (3);

1. Importance of transition being youth focused
2. Emphasis on self-determination, self-management and family/caregiver engagement
3. Acknowledging individual difficulties and complexities
4. Recognizing vulnerabilities and the need for a population health approach
5. Importance of shared accountability and care co-ordination between pediatric and adult centers
6. Recognition of the influence of cultural beliefs, attitudes and socio-economic status
7. Emphasis on achieving health equality, eliminating disparities
8. Parents/caregivers to support young people to develop health knowledge and skills.

The Six Core Elements of Transition 2.0 are elements of a transition that must be present for it to be considered successful (4, 57, 58). These core elements are establishing a transition policy, tracking the transition process, administering transition readiness tools/checklists, planning for, transfer to and integration into adult care (58).

Combinations of these components appear in various forms in the majority of the proposed models for transition. Published models exist for transition in primary care/general pediatric care (for example the Medicaid Managed Care Plan and Health Practice Transformation Model) and for mental health care (European Union-Funded Transition Project) (57). Other models have been published for specific medical conditions, such as sickle cell anemia, renal transplant recipients and type 1 diabetes (57). Again, although the models are different, they all incorporate various aspects of the Six Core Elements of Transition 2.0 and include clinic based transition models, multi-disciplinary team models, transition co-ordinator models, patient developed transition curriculum and web based/mobile health interventions (57). Readers are directed to the excellent and comprehensive review by Wright et al. (57) for discussion and critical evaluation of the various models.

In 2016 a Cochrane review of intervention studies for transition models only included 4 small studies (*n* = 261 patients) and was unable to make conclusions regarding the effectiveness of various models in managing the chronic medical conditions during the transition process, healthcare outcomes or healthcare utilization (59). However, the review by Wright et al. (57) highlights the importance of a nominated individual to act as the “transition co-ordinator” and facilitate the process as a common component of the models that were felt to be the most effective in their review. Although lack of provision of extra staffing and financial incentives for providing transition services has been identified as a barrier to implementing and sustaining transition programs, many of these effective models were able to perform effectively without extra staff or financial incentives (57).

In addition, the needs of a health care service (specifically education and resources) to provide an effective transition program need to be considered prior to choosing and implementing a transition model, based on the suitability of that particular model within the constraints of the health care system.

There are many considerations when choosing a model to use when implementing a transition program, and this highlights the importance of critical evaluation of transition models when determining effectiveness. The lack of published evidence comparing different models, particularly interventional studies, is a barrier to successful implementation of transition models and highlights the need for more research in this area.

## General Considerations for Transition

Table 1 for a summary of issues to be considered during transition. The general focus of pediatric health care can be very different to adult health care. Whereas, pediatric health care tends to be developmentally focused and family centered, development, growth, and family concerns are not commonly primary concerns for adult physicians (56). However, adult health care places an emphasis on autonomy, employment and reproductive health, which are often ignored in pediatric medicine, particularly as pediatricians may struggle with their patient's increased independence, autonomy and “adult” behaviors (56). This struggle of balancing the adolescent's need for increasing autonomy and individuation with the need for adherence to treatment is a struggle not only for pediatricians but parents. Transition can be complicated by a chronic illness as it can be difficult for parents to “let go” and allow the young person to assume primary responsibility for their medical treatment and develop their independence but at the same time ensure adherence to treatment remains optimal and medical management is continued. This struggle is often unconsciously reinforced by the pediatric health care system, which encourages family centered care and is at risk of “infantilising” the young adult (44). This can impede the transition process, particularly as it has been demonstrated that pediatric physicians are often reluctant to “let go” of their patients, and may consciously or unconsciously reinforce the idea that the adult health care system is a frightening environment (44). On the contrary, adult physicians may find it difficult to act in a manner they deem paternalistic when a young adult has not gained the necessary independence to demonstrate autonomy with their medical care, or feel frustrated with receiving patients whom they feel were “mollycoddled” or “treated as babies” by the pediatric system.

**Table 1:** Considerations for transition.

**Considerations for transition**	**Pediatric perspective**	**Adult perspective**	**Organizational perspective**
Preparation for transition (young person and their family)	Start transition process early Normalize the transition process Joint clinics Regular assessment of readiness for transition See the young person on their own Encourage their independence and assuming responsibility for healthcare	Meet the young person prior to formal transition Normalize the transition process Joint clinics Adolescent health screen to build rapport Organize tour of adult site	Encourage joint clinics Consider formalized transition pathway/process Appoint a specific transition co-ordinator Facilitate visit to adult site
Poor adherence to treatment	Open and honest discussion about barriers to poor adherence Problem solving with the young adult Focus on immediate consequences, personal appearance Screening for depression	Open and honest discussion about barriers to poor adherence Problem solving with the young adult Focus on immediate consequences, personal appearance Screening for depression	Consider formalized transition pathway/process Appoint a specific transition co-ordinator
Avoiding being lost to follow-up	Ensure formal transition to adult team with letters and good communication Consider booking a final appointment/phone consult after seeing adult team to ensure everything is in place	Ensure clear communication with pediatric team as to when adult team will be taking over Ensure adult team knows contact details for young person and correct address to send appointment to Consider a written handout about arranging appointments or urgent review	Establish a “safety net”—transition co-ordinator or respiratory nurse to ensure follow-up has occurred
Clinical deterioration during transition process	Ensure follow-up with adult team occurs Address poor adherence as much as possible Delay transition if a major change in clinical status occurs Flag young people with complex issues to inform adult team May require several joint appointments Ensure verbal and written handover for all medical and allied health services Assess medical knowledge and understanding for the young person prior to transition	Close liaison with pediatric team to cover all outstanding issues Be prepared to attend more than one joint appointment	Have a “Transition Checklist” to ensure nothing is missed

Barriers within the healthcare system to implementing a sustainable transition process include lack of infrastructure for co-ordination (particularly at adult healthcare sites), lack of financial reward with increased workload to implement programs and lack of electronic or shareable medical records (58). The difficult with multiple interfaces for entering and storing healthcare information can present a particular challenge, as it can significantly impede communication between pediatric and adult sites. It is useful to have a standard format for transition documents to be prepared by the pediatric service. If regular transitions are occurring to the same adult service, involving the adult physicians in designing a template for exactly which information they need and which formats will be compatible with their record system will reduce the risk of poor handover or loss of medical records.

Engagement of adult physicians in the transition process is essential to ensuring a successful transition, but can present a significant barrier (3). Adult physicians may require a significant amount of education regarding medical conditions that were traditionally considered “pediatric” diseases, with children not surviving to adolescence or adulthood, such as metabolic disorders, cystic fibrosis and congenital heart disease. Adult physicians may understandably feel overwhelmed at providing care to these very complex patients when they may not be familiar with their underlying medical condition, let alone the numerous challenges individuals with these conditions may face in young adulthood. Similarly, a unique challenge exists when there are no equivalent adult services for specific pediatric conditions, particularly those with inborn errors of metabolism, complex neuro-behavioral disorders, cerebral palsy and developmental disorders.

Although some adolescents wish to transition early as they find attending a pediatric center embarrassing, many find transition to be a distressing time due to loss of familiarity, particularly those who have been seen at the same center by the same team for many years. Preparation for transition to adult services should start early and young people and their families should be well-informed of the proposed timeline for the process. The age of transition varies between centers and should be individualized to the young person, however, generally will take place between the ages of 16–18, or when completing secondary education. This is a time of great change in all domains of a young person's life (44). Therefore, a transition checklist is very useful, ensuring all aspects of the transition have been covered (which may include a physical visit to the new site, ensuring the process for obtaining prescriptions is known, how to get to the new appointment and how to seek medical review or emergency review). Particularly for complex patients who require tertiary level care or a dedicated team, having prior appointments with the adult team or even joint appointments prior to transition can be extremely useful to familiarize the young person and their family with the new team and facilities. Young people have reported a tour of the adult facility, joint sessions and a familiar face from the pediatric team joining the first adult appointment are very reassuring (60).

Guidelines and standards for the transition process have been published for a wide range of medical conditions, including diabetes (61), congenital heart disease (62), and cystic fibrosis (63). The transition from being a dependent child to an independent adult is not always smooth, and becomes more complex when there is a chronic medical condition (44). The transition process must be well-planned and must not be rushed, and careful consideration of adolescent issues as well as medical issues must be addressed prior to handing over medical care to an adult physician (56, 64, 65). As well as managing general adolescent issues, transitioning a young person with asthma (particularly severe or poorly controlled asthma) offers some unique challenges. Specific considerations for transitioning the asthmatic patient with to adult heath care services is discussed in more detail below.

## Specific Considerations for Transitioning Patients With Asthma

There is very little published literature specifically examining the transition process in adolescents and young adults with asthma (66). This is surprising given asthma is the most common chronic medical condition in children (5), and highlights the need for further research to determine optimal care for adolescents transition with asthma. For the majority of young people and adolescents with well-controlled asthma, transitioning to adult services is unlikely to be a difficult process. Indeed those already being managed very well in general practice/primary practice do not require transition at all as they can remain with their primary care provider, ensuring excellent continuity. If there is any concern about deterioration of asthma control in young adulthood, an opinion from an adult respiratory specialist could be sought, while the general practitioner maintains primary management with specialist reviews as required.

Those well-managed by general pediatricians are likely to be able to transition to ongoing care in general practice, and liaison with a suitable general practitioner should be encouraged early on to ensure they are comfortable with the transition plan. A study of the transition process for adolescents with asthma at a children's hospital asthma/allergy clinic found that young people with mild/moderate asthma were managed equally effectively regardless of whether they transitioned to primary or specialist care (66). All patients in this study with severe asthma were transitioned to specialist adult asthma services (66). It is important that adolescents with asthma do have ongoing follow-up, even if their asthma is mild, as childhood asthma is a risk factor for developing chronic obstructive pulmonary disease (COPD) in later life (27, 36, 37). In addition, asthma can recur in adult life despite remission as a teenager (30).

Adolescents who require tertiary level care are more likely to have difficult to control or severe asthma and may have other considerations which complicate the transition process (particularly poor adherence to treatment, exposure to cigarette smoke, mental health concerns or additional medical conditions). These complicating factors are common in asthmatic adolescents. For example, in a study of adolescents with asthma, 25% were reported to be poorly adherent to treatment (66). Of particular concern is adolescents with asthma who smoke or are exposed to passive smoke from peers. A study of Swedish adolescents with asthma found that 8% reported being smokers and 28% were exposed to passive smoke at home or by their peers (66). The incidence of depression in individuals with asthma is higher than the general population and depression in asthmatics may be more common than in other chronic medical conditions (67). In addition, depression may increase morbidity and mortality associated with asthma (67). These concerns that may complicate transition should be identified and managed if possible prior to transition and handed over to the adult team for ongoing management.

The vast majority of adolescents requiring tertiary level care for their asthma would be expected to require tertiary level respiratory care as an adult, especially those requiring frequent hospital and/or intensive care admissions. This is reinforced in the study above, where all adolescents with severe asthma required transition to specialist adult respiratory services (66). If a tertiary adult respiratory service is not available, an adult respiratory physician with an interest in asthma could be considered to take over ongoing care. In more remote locations visiting respiratory services may be available, with a local doctor taking primary responsibility for ongoing care. Young people who are likely to require ongoing hospital admissions for exacerbations of asthma should be referred to a service that is able to manage emergency presentations, admissions and provide frequent review. In these cases, a suitable service with experience in managing difficult asthma should be identified early on and liaison should occur prior to transition, particularly if there is the possibility of a joint appointment or meeting the adult team prior to transition. Development of a specific center-based transition pathway for adolescents with difficult or severe asthma is encouraged to formalize the process and ensure nothing is missed.

Adolescent and young adult patients with frequent asthma exacerbations requiring presentation and management in emergency departments present additional challenges during the transition period. A review of care transition within the emergency department back to primary care identified that those who presented to ED often received improper management of exacerbations at home, and families required education and support to transition safely back to primary care (5). Although these findings are presented in the context of the emergency department, they highlight the need for excellent communication, care coordination and patient education to ensure a successful transition, regardless of the health care providers involved. It also highlights the need to specifically consider the high care needs of asthmatic patients who are frequent presenters at emergency services, and the value of involving the emergency department in planning for transition and ensuring access to adult emergency services as part of preparing for the transition process.

A common concern in chronic medical conditions is deterioration of stability during the transition process and loss to follow-up by adult services (44). This may delay the transition process, particularly if the pediatric service is not confident the young adult has acquired the skills to manage their medical condition or seek help appropriately if unwell. Bergstrom et al. (66) studied 150 adolescents with asthma during the transition process to identify risk factors for deterioration of asthma control during and after transition. They found that it was rare for pulmonary function to deteriorate during and up to 5 years post transition (66). In addition, the majority of those with reduced FEV1 at the time of transition had improved pulmonary function at follow-up (66) which is encouraging. Poor adherence to medical treatment and female gender predicted persisting bronchial hyper responsiveness and chronic symptoms (66), therefore this groups of patients may require more frequent monitoring during and post transition.

Untreated or poorly controlled asthma can be associated with pubertal delay (68–71), and the associated emotional distress secondary to pubertal delay can contribute to poorer asthma control and poor adherence to treatment (68). This is particularly the case as there is some evidence inhaled corticosteroids may contribute to pubertal delay (69) hence adolescent patients may be reluctant to use them. However, untreated or poorly treated asthma delays puberty on average by 1.3 years (71) and discussing this information may encourage adherence to treatment. Many young people with asthma are concerned about short stature and may attribute this to chronic use of inhaled corticosteroids, however in severe or poorly controlled asthma pubertal delay itself is more likely to explain the apparent growth failure rather than suppression from corticosteroids (71). Reassurance that treating asthma and better adherence to treatment may improve the pubertal delay and short stature may assist in encouraging adherence to treatment. Pubertal status and stature should be monitored closely in all adolescent patients with asthma, particularly those on high dose inhaled corticosteroids or oral steroids.

Prior to transition, contraception and sexual history should always be discussed. Although the majority of detailed discussion will be with female patients, sexual history should also be discussed with males and an open discussion about which form of contraception they use is encouraged. When choosing contraception to avoid unwanted pregnancy in asthmatic adolescents, a number of considerations are necessary. Firstly, barrier contraception is the only method offering any protection against sexually transmitted diseases, and barrier contraceptives should be used in combination with a more effective form of contraception for avoiding pregnancy. Second, if adherence to treatment is a challenge, a reliable form of contraception that doesn't rely on remembering to take medication should be chosen, such as a hormonal implant or intra-uterine device. In particular the Progesterone Only Pill should be avoided as efficacy is dependent upon taking it regularly and at the same time each day. Thirdly, contraception should be encouraged, particularly in severe asthmatics, as asthma exacerbations during pregnancy have been shown to be associated with low birthweight babies (72) and oral contraception use in pregnancy has been shown to reduce the risk of asthma exacerbations (73). Although preconception and obstetric care of the asthmatic women is beyond the scope of this article, readers are directed to resources such as the Australian Asthma Handbook section on pregnancy and preconception considerations (74) (available from http://www.asthmahandbook.org.au). Although there appears to be a significant association between maternal ingestion of Paracetamol during pregnancy and subsequent risk of asthma in offspring (75), non-causal explanations have not been able to be excluded (76), and a significant number of confounders exist, particularly family history of asthma and atopy. The large Norwegian Mother and Child Cohort Study found that prenatal exposure to Paracetamol was associated with a small increase (13%) in the risk of developing asthma when accounting for confounders (77). Given infants of asthmatic mothers will already be at a higher risk of developing asthma given family history of asthma and/or atopy, it may be prudent to advise adolescent and young adult women of childbearing age to avoid Paracetamol while pregnant or planning a pregnancy.

Financial concerns after transition are more likely to be a concern for young adults, as adolescents will often live in the family home. Scal et al. (78) found that adolescents with asthma were more likely to have a usual care provider and private health insurance than young adults with asthma, with young adults facing increased financial barriers to accessing health care after transition. Of concern, these young adults reported more unmet health care needs and delays in receiving health care due to financial constraints (78). Other barriers to accessing appropriate health care for asthma in both adolescents undergoing transition and young adults post transition were difficulties with transportation, frustration at long wait times at physician offices, difficulties arranging appointments (especially by telephone) and inconvenient physician office hours (78). Addressing these concerns may require social work involvement and provision of financial assistance to ensure continued access to health care post transition.

Gibson-Scipio et al. (79) highlighted the additional challenges for young adults with asthma who are transitioning and are not Caucasian. For example, it is known that asthma morbidity and mortality rates are higher in those individuals who are not Caucasian (80). In addition, African American youth are less likely to discuss transition or engage in the transition process than Caucasian youth (81). In their study, Gibson-Scipio et al. (79) found that African American youth with asthma often did not use preventer medications and lacked understanding of the inflammatory nature of asthma that required preventer medication even when symptoms were well-controlled. Nearly half expressed that they didn't want to inform their peers of their medical condition as it was a sign of “weakness” and most felt routine visits for asthma were unnecessary (79). Although these concerns are unlikely to be limited to adolescents who are not Caucasian, it raises the importance of ensuring transition is well-planned and there is a good understanding of the medical condition, particularly in those groups with a higher risk of morbidity and poor ongoing engagement.

The importance of health education and assessing understanding of the underlying medical condition cannot be overstated, particularly when preparing for transition. Although this is important for all patients with asthma, it is particularly important in non-Caucasian adolescents who are at higher risk of poorer health outcomes and poor understanding of the their asthma and treatments (79, 82). Holley et al. (83) demonstrated that understanding of the medical condition and treatments was essential for allowing adolescents to develop self-management skills and improve adherence to treatment. Lack of knowledge about asthma was identified by adolescents as a significant barrier to adherence to treatment (83). Particularly important focuses for education were triggers, how to recognize symptoms indicating an exacerbation and the seriousness of asthma (83). Other groups describe the importance of educating about treatments, how they work and why to use them as key to encouraging self-management of asthma by adolescent patients (83–85). Correct inhaler technique is essential, particularly as few children use their inhalers in the correct way (86). Volerman et al. (86) highlight the need for careful assessment and direct observation of inhaler technique, given parents and children will over-estimate their skills in delivering inhaled medications effectively. If poor inhaler technique can be corrected prior to transition, it may contribute to better inhaler technique in adult patients, as up to 90% of adults with asthma and COPD do not use their inhalers correctly (87, 88). It is essential that people with asthma carry their inhalers at all times, and for young people there can be barriers to carrying their inhalers and using them at school (89), such poor knowledge of how to assemble the inhaler in an emergency (90). These results emphasize the importance of practical asthma education including careful assessment of inhaler technique, assembly of devices and discussing practical strategies for carrying devices at school to ensure rescue medications are used effectively. Other strategies for education written action plans for daily management (very common in pediatrics), formal asthma education with an asthma nurse and partnering with schools and housing authorities (82). Transition presents an opportunity for assessment of asthma knowledge, particularly treatments and identifying exacerbations, and encouraging the adolescent to be able to self-manage in preparation for transition. Importantly, it has been demonstrated that health education regarding asthma treatment can improve remove health literacy barriers, improving asthma self-management and treatment adherence (91). Therefore, the importance of regular asthma education with a focus on practical assessment prior to and after transition (and on an ongoing basis in adult health care) cannot be overstated.

## A Suggested Approach Following Transition to Adult Services

For those young adults who do require transition to specialist adult asthma services, the transfer of their care provides an opportunity for a detailed review of their asthma, starting with a careful reassessment of the diagnosis. For most patients a review of the pediatric case notes will identify objective evidence supporting the diagnosis, for example presence of variable airflow obstruction and/or airway hyper responsiveness, often along with markers of airway inflammation such as elevated FeNO, blood or airway eosinophilia. In the absence of this evidence, or where the pattern of symptoms or exacerbations has changed, it may be helpful to organize further investigations such as methacholine or histamine challenge testing, induced sputum, FeNO, and in selected cases to consider CT imaging or bronchoscopy. These investigations will not only help to confirm that asthma is still the primary condition driving their morbidity, but also help to characterize the underlying asthma phenotype and to determine which are the active treatable traits that require attention (11, 42, 43). Identification of an eosinophilic inflammation predominant phenotype, for example, may direct the clinician to consider targeted anti-eosinophil therapies such as anti-IL5 treatment (44, 45). As adolescent patients transfer to adult services and start to take more individual responsibility for their own healthcare, non-adherence to treatment is a particularly important issue (46). Transfer to adult services should prompt a full medication review including an assessment of the patients' beliefs and understanding of the rationale for treatment, their current and anticipated adherence pattern, use of rescue medication including any inappropriate reliance on short acting B2 agonists, inhaler technique and presence and understanding of a personalized asthma action plan for exacerbations. A review of inhaler device and treatment doses will often also be appropriate. Similarly it is important to remember that patients are often undergoing significant changes in their psychosocial circumstances such moving away to university or starting full time employment and it will be important to consider the impact that these lifestyle changes will have on the patient's asthma. With appropriate initial support from the multidisciplinary asthma team most patients can accept increasing responsibility for managing their own asthma following transition. For some young adults, particularly those with the most severe asthma who have had a history of frequent hospital admissions, more time will be needed to lessen the need for the input of parents and other carers, but wherever possible the goal should be independent asthma self-management with support from the specialist asthma team.

## Author Contributions

All authors listed have made a substantial, direct and intellectual contribution to the work, and approved it for publication.

### Conflict of Interest Statement

The authors declare that the research was conducted in the absence of any commercial or financial relationships that could be construed as a potential conflict of interest.

## References

[1] Blum RW Garell D Hodgman CH Jorissen TW Okinow NA Orr DP Transition from child-centred care to adult health-care systems for adolescents with chronic conditions. A position paper of the Society for Adolescent Medicine. J Adolesc Health. (1993) 14:570–6. 10.1016/1054-139X(93)90143-D 8312295

[2] White PH Cooley WC Supporting the health care transition from adolescence to adulthood in the medical home. Pediatrics. (2011) 128:182–200. 10.1542/peds.2011-0969 21708806

[3] White PH Cooley WC. Supporting the health care transition from adolescence to adulthood in the medical home. Pediatrics. (2018) 142:e20182587. 10.1542/peds.2018-2587 30348754

[4] White PH Cooley WC Supporting the health care transition from adolescence to adulthood in the medical home. *Pediatrics*. (2018) 142:e20182587 Pediatrics. (2019) 143:e20183610 10.1542/peds.2018-3610 30348754

[5] Martin MA Press VG Nyenhuis SM Krishnan JA Erwin K Mosnaim G. Care transition interventions for children with asthma in the emergency department. J Allergy Clin Immunol. (2016) 138:1518–25. 10.1016/j.jaci.2016.10.012 27931533 PMC5327498

[6] Fuchs O Bahmer T Rabe KF von Mutius E. Asthma transition from childhood into adulthood. Lancet Respir Med. (2017) 5:224–34. 10.1016/S2213-2600(16)30187-4 27666650

[7] Devulapalli CS Carlsen KC Haland G Munthe-Kaas MC Pettersen M Mowinckel P. Severity of obstructive airways disease by age 2 years predicts asthma at 10 years of age. Thorax. (2008) 63:8–13. 10.1136/thx.2006.060616 17615086

[8] Bush A Menzies-Gow A. Phenotypic differences between pediatric and adult asthma. Proc Am Thorac Soc. (2009) 6:712–9. 10.1513/pats.200906-046DP 20008882

[9] Depner M Fuchs O Genuneit J Karvonen AM Hyvarinen A Kaulek V. Clinical and epidemiologic phenotypes of childhood asthma. Am J Respir Crit Care Med. (2014) 189:129–38. 10.1164/rccm.201307-1198OC 24283801

[10] Henderson J Granell R Heron J Sherriff A Simpson A Woodcock A. Associations of wheezing phenotypes in the first 6 years of life with atopy, lung function and airway responsiveness in mid-childhood. Thorax. (2008) 63:974–80. 10.1136/thx.2007.093187 18678704 PMC2582336

[11] Stern DA Morgan WJ Halonen M Wright AL. Wheezing and bronchial hyper-responsiveness in early childhood as predictors of newly diagnosed asthma in early adulthood: a longitudinal birth-cohort study. Lancet. (2008) 372:1058–64. 10.1016/S0140-6736(08)61447-6 18805334 PMC2831297

[12] Albuali WH Singh RN Fraser DD Seabrook JA Kavanagh BP Parshuram CS. Have changes in ventilation practice improved outcome in children with acute lung injury? Pediatr Crit Care Med. (2007) 8:324–30. 10.1097/01.PCC.0000269390.48450.AF 17545937

[13] Xuan W Marks GB Toelle BG Belousova E Peat JK Berry G. Risk factors for onset and remission of atopy, wheeze, and airway hyperresponsiveness. Thorax. (2002) 57:104–9. 10.1136/thorax.57.2.104 11828037 PMC1746247

[14] Russell G. Asthma in the transition from childhood to adulthood. Thorax. (2002) 57:96–7. 10.1136/thorax.57.2.96 11828035 PMC1746241

[15] Kelly WJ Hudson I Phelan PD. Childhood asthma in adult life: a further study at 28 years of age. BMJ. (1987) 294:1059–62. 10.1136/bmj.294.6579.1059 3107692 PMC1246220

[16] Phelan PD. Hyperresponsiveness as a determinant of the outcome in childhood asthma. Am Rev Respir Dis. (1991):1463–6; discussion 6–7. 10.1164/ajrccm/143.6.1463 2048839

[17] Roorda RJ Gerritsen J Van Aalderen WM Schouten JP Veltman JC Weiss ST. Risk factors for the persistence of respiratory symptoms in childhood asthma. Am Rev Respir Dis. (1993) 148 (6 Pt 1):1490–5. 10.1164/ajrccm/148.6_Pt_1.1490 8256889

[18] Ulrik CS Backer V Hesse B Dirksen A. Risk factors for development of asthma in children and adolescents: findings from a longitudinal population study. Respir Med. (1996) 90:623–30. 10.1016/S0954-6111(96)90021-9 8959120

[19] Fagan JK Scheff PA Hryhorczuk D Ramakrishnan V Ross M Persky V. Prevalence of asthma and other allergic diseases in an adolescent population: association with gender and race. Ann Allergy Asthma Immunol. (2001) 86:177–84. 10.1016/S1081-1206(10)62688-9 11258687

[20] de Marco R Locatelli F Sunyer J Burney P. Differences in incidence of reported asthma related to age in men and women. A retrospective analysis of the data of the European Respiratory Health Survey. Am J Respir Crit Care Med. (2000) 162:68–74. 10.1164/ajrccm.162.1.9907008 10903222

[21] Martin AJ Landau LI Phelan PD. Natural history of allergy in asthmatic children followed to adult life. Med J Aust. (1981) 2:470–4. 7321996 10.5694/j.1326-5377.1981.tb112942.x

[22] Blair H. Natural history of childhood asthma. 20-year follow-up. Arch Dis Child. (1977) 52:613–9. 10.1136/adc.52.8.613 921306 PMC1544640

[23] Cserhati E Mezei G Kelemen J. Late prognosis of bronchial asthma in children. Respiration. (1984) 46:160–5. 10.1159/000194685 6494612

[24] Strachan DP Butland BK Anderson HR. Incidence and prognosis of asthma and wheezing illness from early childhood to age 33 in a national British cohort. BMJ. (1996) 312:1195–9. 10.1136/bmj.312.7040.1195 8634562 PMC2350975

[25] Jenkins MA Hopper JL Bowes G Carlin JB Flander LB Giles GG. Factors in childhood as predictors of asthma in adult life. BMJ. (1994) 309:90–3. 10.1136/bmj.309.6947.90 8038673 PMC2540556

[26] Vonk JM Postma DS Boezen HM Grol MH Schouten JP Koeter GH. Childhood factors associated with asthma remission after 30 year follow up. Thorax. (2004) 59:925–9. 10.1136/thx.2003.016246 15516465 PMC1746857

[27] Sears MR Greene JM Willan AR Wiecek EM Taylor DR Flannery EM. A longitudinal, population-based, cohort study of childhood asthma followed to adulthood. N Engl J Med. (2003) 349:1414–22. 10.1056/NEJMoa022363 14534334

[28] Tai A Tran H Roberts M Clarke N Gibson AM Vidmar S. Outcomes of childhood asthma to the age of 50 years. J Allergy Clin Immunol. (2014) 133:1572–8 e3. 10.1016/j.jaci.2013.12.1033 24495434

[29] Andersson M Hedman L Bjerg A Forsberg B Lundback B Ronmark E. Remission and persistence of asthma followed from 7 to 19 years of age. Pediatrics. (2013) 132:e435–42. 10.1542/peds.2013-0741 23897917

[30] Taylor DR Cowan JO Greene JM Willan AR Sears MR. Asthma in remission: can relapse in early adulthood be predicted at 18 years of age? Chest. (2005) 127:845–50. 10.1378/chest.127.3.845 15764766

[31] Sood A Qualls C Schuyler M Arynchyn A Alvarado JH Smith LJ. Adult-onset asthma becomes the dominant phenotype among women by age 40 years. The longitudinal CARDIA study. Ann Am Thorac Soc. (2013) 10:188–97. 10.1513/AnnalsATS.201212-115OC 23802814 PMC3960903

[32] Burgess JA Walters EH Byrnes GB Giles GG Jenkins MA Abramson MJ. Childhood adiposity predicts adult-onset current asthma in females: a 25-yr prospective study. Eur Respir J. (2007) 29:668–75. 10.1183/09031936.00080906 17251231

[33] Castro-Rodriguez JA Holberg CJ Morgan WJ Wright AL Martinez FD Increased incidence of asthmalike symptoms in girls who become overweight or obese during the school years. Am J Respir Crit Care Med. (2001) 163:1344–9. 10.1164/ajrccm.163.6.2006140 11371399

[34] Spycher BD Kuehni CE. Asthma phenotypes in childhood: conceptual thoughts on stability and transition. Eur Respir J. (2016) 47:362–5. 10.1183/13993003.02011-2015 26828043

[35] Hirose M Horiguchi T. Asthma phenotypes. J Gen Fam Med. (2017) 18:189–94. 10.1002/jgf2.7 29264025 PMC5689426

[36] Vonk JM Jongepier H Panhuysen CI Schouten JP Bleecker ER Postma DS. Risk factors associated with the presence of irreversible airflow limitation and reduced transfer coefficient in patients with asthma after 26 years of follow up. Thorax. (2003) 58:322–7. 10.1136/thorax.58.4.322 12668795 PMC1746641

[37] de Marco R Marcon A Jarvis D Accordini S Almar E Bugiani M. Prognostic factors of asthma severity: a 9-year international prospective cohort study. J Allergy Clin Immunol. (2006) 117:1249–56. 10.1016/j.jaci.2006.03.019 16750983

[38] McGeachie MJ Yates KP Zhou X Guo F Sternberg AL Van Natta ML. Patterns of growth and decline in lung function in persistent childhood asthma. N Engl J Med. (2016) 374:1842–52. 10.1056/NEJMoa1513737 27168434 PMC5032024

[39] Gupta A Bazari F Holloway E Bossley C Payne D Wilson N Progression of paediatric difficult asthma five years after initial assessment. Am J Respir Crit Care Med. (2009) 179:a4840 10.1164/ajrccm-conference.2009.179.1_MeetingAbstracts.A4840

[40] Nordlund B Melen E Schultz ES Gronlund H Hedlin G Kull I. Prevalence of severe childhood asthma according to the WHO. Respir Med. (2014) 108:1234–7. 10.1016/j.rmed.2014.05.015 24939389

[41] Guilbert TW Bacharier LB Fitzpatrick AM. Severe asthma in children. J Allergy Clin Immunol Pract. (2014) 2:489–500. 10.1016/j.jaip.2014.06.022 25213041 PMC4589165

[42] Plaza-Martin AM Vennera MC Galera J Herraez L Group PS. Prevalence and clinical profile of difficult-to-control severe asthma in children: results from pneumology and allergy hospital units in Spain. Allergol Immunopathol. (2014) 42:510–7. 10.1016/j.aller.2014.02.003 24948187

[43] Garner R Kohen D. Changes in the prevalence of asthma among Canadian children. Health Rep. (2008) 19:45–50. 18642518

[44] Withers AL. Management issues for adolescents with cystic fibrosis. Pulm Med. (2012) 2012:134132. 10.1155/2012/134132 22991662 PMC3444048

[45] Zack J Jacobs CP Keenan PM Harney K Woods ER Colin AA. Perspectives of patients with cystic fibrosis on preventive counseling and transition to adult care. Pediatr Pulmonol. (2003) 36:376–83. 10.1002/ppul.10342 14520719

[46] Segal TY. Adolescence: what the cystic fibrosis team needs to know. J R Soc Med. (2008) 101 (Suppl. 1):S15–27. 10.1258/jrsm.2008.s18005 18607014 PMC2443986

[47] Suris JC Parera N. Sex, drugs and chronic illness: health behaviours among chronically ill youth. Eur J Public Health. (2005) 15:484–8. 10.1093/eurpub/cki001 16162596

[48] Quittner AL Barker DH Snell C Grimley ME Marciel K Cruz I. Prevalence and impact of depression in cystic fibrosis. Curr Opin Pulm Med. (2008) 14:582–8. 10.1097/MCP.0b013e3283121cf1 18812836

[49] Bhatia SK Bhatia SC. Childhood and adolescent depression. Am Fam Physician. (2007) 75:73–80. 17225707

[50] Evans DL Charney DS Lewis L Golden RN Gorman JM Krishnan KR. Mood disorders in the medically ill: scientific review and recommendations. Biol Psychiatry. (2005) 58:175–89. 10.1016/j.biopsych.2005.05.001 16084838

[51] Cadman D Boyle M Szatmari P Offord DR. Chronic illness, disability, and mental and social well-being: findings of the Ontario Child Health Study. Pediatrics. (1987) 79:805–13. 2952939

[52] Greydanus D Patel D Pratt H. Suicide risk in adolescents with chronic illness: implications for primary care and specialty pediatric practice: a review. Dev Med Child Neurol. (2010) 52:1083–7. 10.1111/j.1469-8749.2010.03771.x 20813018

[53] Suris JC Parera N Puig C. Chronic illness and emotional distress in adolescence. J Adolesc Health. (1996) 19:153–6. 10.1016/1054-139X(95)00231-G 8863088

[54] Fiese BH Everhart RS. Medical adherence and childhood chronic illness: family daily management skills and emotional climate as emerging contributors. Curr Opin Pediatr. (2006) 18:551–7. 10.1097/01.mop.0000245357.68207.9b 16969171

[55] Helgeson VS Novak SA. Illness centrality and well-being among male and female early adolescents with diabetes. J Pediatr Psychol. (2007) 32:260–72. 10.1093/jpepsy/jsl018 16837739

[56] Viner R. Transition from paediatric to adult care. Bridging the gaps or passing the buck? Arch Dis Child. (1999) 81:271–5. 10.1136/adc.81.3.271 10451404 PMC1718076

[57] Wright C Steinway C Jan S. The genesis of systems of care for transition to adulthood services: emerging models in primary and subspecialty care. Curr Opin Pediatr. (2018) 30:303–10. 10.1097/MOP.0000000000000608 29406441

[58] McManus M White P Pirtle R Hancock C Ablan M Corona-Parra R. Incorporating the six core elements of health care transition into a medicaid managed care plan: lessons learned from a pilot project. J Pediatr Nurs. (2015) 30:700–13. 10.1016/j.pedn.2015.05.029 26239121

[59] Campbell F Biggs K Aldiss SK O'Neill PM Clowes M McDonagh J. Transition of care for adolescents from paediatric services to adult health services. Cochrane Database Syst Rev. (2016) 4:CD009794. 10.1002/14651858.CD009794.pub2 27128768 PMC10461324

[60] Brumfield K Lansbury G Experiences of adolescents wtih cystic fibrosis durings teir transition from paediatric to adult health care: a qualitative study of young Australian adults. Disabil Rehab. (2004) 26:223–34. 10.1080/09638280310001644924 15164956

[61] Nakhla M Bell LE Wafa S Dasgupta K. Improving the transition from pediatric to adult diabetes care: the pediatric care provider's perspective in Quebec, Canada. BMJ Open Diabetes Res Care. (2017) 5:e000390. 10.1136/bmjdrc-2017-000390 28761657 PMC5530239

[62] Sable C Foster E Uzark K Bjornsen K Canobbio MM Connolly HM. Best practices in managing transition to adulthood for adolescents with congenital heart disease: the transition process and medical and psychosocial issues: a scientific statement from the American Heart Association. Circulation. (2011) 123:1454–85. 10.1161/CIR.0b013e3182107c56 21357825

[63] Al-Yateem N. Guidelines for the transition from child to adult cystic fibrosis care. Nurs Child Young People. (2013) 25:29–34. 10.7748/ncyp2013.06.25.5.29.e175 23988074

[64] Couriel J. Asthma in adolescence. Paediatr Respir Rev. (2003) 4:47–54. 10.1016/S1526-0542(02)00309-3 12615032

[65] American Academy of Pediatrics, American Academy of Family Physicians, American College of Physicians-American Society of Internal Medicine A consensus statement on health care transitions for young adults with special health care needs. Pediatrics. (2002) 110 (6 Pt 2):1304–6. 12456949

[66] Bergstrom SE Sundell K Hedlin G. Adolescents with asthma: consequences of transition from paediatric to adult healthcare. Respir Med. (2010) 104:180–7. 10.1016/j.rmed.2009.09.021 19889523

[67] Zielinski TA Brown ES Nejtek VA Khan DA Moore JJ Rush AJ. Depression in asthma: prevalence and clinical implications. Prim Care Companion J Clin Psychiatry. (2000) 2:153–8. 10.4088/PCC.v02n0501 15014636 PMC181132

[68] Balfour-Lynn L. Effect of asthma on growth and puberty. Pediatrician. (1987) 14:237–41. 3331205

[69] Dakhel AKA Alqeaid FAR Alkhuzayyim FMA Association between bronchial asthma and pubertal delay in pediatric patients. Egypt J Hospital Med. (2018) 70:245–50. 10.12816/0043084

[70] Wolthers OD. Growth problems in children with asthma. Horm Res. (2002) 57 (Suppl. 2):83–7. 10.1159/000058107 12065934

[71] Doull IJ. The effect of asthma and its treatment on growth. Arch Dis Child. (2004) 89:60–3. 10.1136/adc.2003.014365 14709510 PMC1755898

[72] Murphy VE Clifton VL Gibson PG. Asthma exacerbations during pregnancy: incidence and association with adverse pregnancy outcomes. Thorax. (2006) 61:169–76. 10.1136/thx.2005.049718 16443708 PMC2104591

[73] Nwaru BI Sheikh A. Hormonal contraceptives and asthma in women of reproductive age: analysis of data from serial national Scottish Health Surveys. J R Soc Med. (2015) 108:358–71. 10.1177/0141076815588320 26152676 PMC4582260

[74] NAC Australian Asthma Handbook, Version 2.0. Melbourne, VIC: NAC (2019).

[75] Fan G Wang B Liu C Li D. Prenatal paracetamol use and asthma in childhood: a systematic review and meta-analysis. Allergol Immunopathol. (2017) 45:528–33. 10.1016/j.aller.2016.10.014 28237129

[76] Andersen AB Farkas DK Mehnert F Ehrenstein V Erichsen R. Use of prescription paracetamol during pregnancy and risk of asthma in children: a population-based Danish cohort study. Clin Epidemiol. (2012) 4:33–40. 10.2147/CLEP.S28312 22355259 PMC4614522

[77] Magnus MC Karlstad O Haberg SE Nafstad P Davey Smith G Nystad W. Prenatal and infant paracetamol exposure and development of asthma: the Norwegian Mother and Child Cohort Study. Int J Epidemiol. (2016) 45:512–22. 10.1093/ije/dyv366 26861478 PMC5841883

[78] Scal P Davern M Ireland M Park K. Transition to adulthood: delays and unmet needs among adolescents and young adults with asthma. J Pediatr. (2008) 152:471–75.e1. 10.1016/j.jpeds.2007.10.004 18346498 PMC3189852

[79] Gibson-Scipio W Gourdin D Krouse HJ. Asthma self-management goals, beliefs and behaviors of Urban African American adolescents prior to transitioning to adult health care. J Pediatr Nurs. (2015) 30:e53–61. 10.1016/j.pedn.2015.06.012 26169338

[80] Lara M Akinbami L Flores G Morgenstern H. Heterogeneity of childhood asthma among Hispanic children: Puerto Rican children bear a disproportionate burden. Pediatrics. (2006) 117:43–53. 10.1542/peds.2004-1714 16396859

[81] Lotstein DS Kuo AA Strickland B Tait F. The transition to adult health care for youth with special health care needs: do racial and ethnic disparities exist? Pediatrics. (2010) 126 (Suppl. 3):S129–36. 10.1542/peds.2010-1466F 21123475

[82] Volerman A Chin MH Press VG. Solutions for asthma disparities. Pediatrics. (2017) 139:e20162546. 10.1542/peds.2016-2546 28228500 PMC5330398

[83] Holley S Walker D Knibb R Latter S Liossi C Mitchell F. Barriers and facilitators to self-management of asthma in adolescents: an interview study to inform development of a novel intervention. Clin Exp Allergy. (2018) 48:944–56. 10.1111/cea.13141 29573024

[84] Rhee H Belyea MJ Ciurzynski S Brasch J. Barriers to asthma self-management in adolescents: relationships to psychosocial factors. Pediatr Pulmonol. (2009) 44:183–91. 10.1002/ppul.20972 19142893 PMC2692882

[85] Buston KM Wood SF. Non-compliance amongst adolescents with asthma: listening to what they tell us about self-management. Fam Pract. (2000) 17:134–8. 10.1093/fampra/17.2.134 10758075

[86] Volerman A Toups MM Hull A Press VG. Does inhaler technique align with confidence among African-American children and their parents? Ann Allergy Asthma Immunol. (2019) 123:100–1. 10.1016/j.anai.2019.04.012 31051238 PMC6599723

[87] Cochrane MG Bala MV Downs KE Mauskopf J Ben-Joseph RH. Inhaled corticosteroids for asthma therapy: patient compliance, devices, and inhalation technique. Chest. (2000) 117:542–50. 10.1378/chest.117.2.542 10669701

[88] Lavorini F Magnan A Dubus JC Voshaar T Corbetta L Broeders M. Effect of incorrect use of dry powder inhalers on management of patients with asthma and COPD. Respir Med. (2008) 102:593–604. 10.1016/j.rmed.2007.11.003 18083019

[89] Toups MM Press VG Volerman A. National analysis of state health policies on students' right to self-carry and self-administer asthma inhalers at school. J Sch Health. (2018) 88:776–84. 10.1111/josh.12681 30203483

[90] Sridharan G Spalding A Press VG Volerman A Barriers and facilitators to self-carry of inhalers in school: a qualitative study of children with asthma. Conference Abstract 195 A:3327. Am J Respir Crit Care Med. (2017) 195.

[91] Paasche-Orlow MK Riekert KA Bilderback A Chanmugam A Hill P Rand CS. Tailored education may reduce health literacy disparities in asthma self-management. Am J Respir Crit Care Med. (2005) 172:980–6. 10.1164/rccm.200409-1291OC 16081544 PMC2718412

